# Cefiderocol-Based Combination Therapy for “Difficult-to-Treat” Gram-Negative Severe Infections: Real-Life Case Series and Future Perspectives

**DOI:** 10.3390/antibiotics10060652

**Published:** 2021-05-29

**Authors:** Davide Fiore Bavaro, Alessandra Belati, Lucia Diella, Monica Stufano, Federica Romanelli, Luca Scalone, Stefania Stolfa, Luigi Ronga, Leonarda Maurmo, Maria Dell’Aera, Adriana Mosca, Lidia Dalfino, Salvatore Grasso, Annalisa Saracino

**Affiliations:** 1 Clinic of Infectious Diseases, Department of Biomedical Sciences and Human Oncology, University of Bari “Aldo Moro”, 70124 Bari, Italy; alessandra.belati@hotmail.it (A.B.); diella.lucia@libero.it (L.D.); annalisa.saracino@uniba.it (A.S.); 2 Anesthesia and Intensive Care Unit, Department of Emergency and Organ Transplantation, University of Bari, 70124 Bari, Italy; monistufa@gmail.com (M.S.); lidia.dalfino@yahoo.com (L.D.); salvatore.grasso@uniba.it (S.G.); 3 Section of Microbiology and Virology, University of Bari, 70124 Bari, Italy; federicarosaromanelli@gmail.com (F.R.); stolfastefania@gmail.com (S.S.); rongalu@yahoo.it (L.R.); adriana.mosca@uniba.it (A.M.); 4 Segreteria Scientifica Comitato Etico Area 2, University of Bari, 70124 Bari, Italy; l.scalo91@virgilio.it (L.S.); leamaurmo94@gmail.com (L.M.); 5 Direttore Farmacia Ospedaliera AOU Policlinico di Bari, University of Bari, 70124 Bari, Italy; maria.dellaera@policlinico.ba.it

**Keywords:** cefiderocol, multidrug resistant gram-negative bacteria, novel antimicrobial strategies, *Pseudomonas aeruginosa*, *Acinetobacter baumannii*, *Klebsiella pneumoniae*, immunocompromised hosts, critically ill patients

## Abstract

Cefiderocol is a new cephalosporin displaying against extensively resistant (XDR) Gram-negative bacteria. We report our experience with cefiderocol-based combination therapies as “rescue” treatments in immunocompromised or critically ill patients or in patients with post-surgical infections who had failed previous regimens. A total of 13 patients were treated from 1 September 2020 to 31 March 2021. In total, 5/13 (38%) patients were classified as critically ill, due to severe COVID-19 lung failure; 4/13 (31%) patients had post-surgical infections and 4/13 (31%) had severe infections in immunocompromised subjects due to solid organ transplantation (2/4) or hematological malignancy (2/4). Overall, 10/13 infections were caused by carbapenem-resistant *Acinetobacter baumannii*, one by KPC-positive ceftazidime/avibactam-resistant *Klebsiella pneumonia* and two by *Pseudomonas aeruginosa* XDR. Based on clinical, microbiological and hematobiochemical evaluation, cefiderocol was associated with different companion drugs, particularly with fosfomycin, high-dose tigecycline and/or colistin. Microbiological eradication was achieved in all cases and the 30-day survival rate was 10/13; two patients died due to SARS-CoV-2 lung failure, whereas one death was attributed to subsequent infections. No recurrent infections within 30 days were reported. Finally, we hereby discuss the therapeutic potential of cefiderocol and the possible place in the therapy of this novel drug.

## 1. Introduction

Cefiderocol (formerly S-649266) is a new generation siderophore cephalosporin which inhibits bacterial wall synthesis, utilizing a “Trojan horse” mechanism based on iron active transporters. It has been developed to be active against extensively resistant (XDR) Gram-negative bacteria (GNB), including carbapenemase-producing Enterobacterales (CPE) and non-fermentative GNB [1]. These pathogens are often involved in difficult-to-treat (DTT) healthcare-associated infections (HCAI) [2], such as ventilator-associated pneumonia (VAP), [3] bloodstream infections (BSIs) [4] and intra-abdominal infections (IAIs) [5], and are burdened by elevated rates of morbidity and mortality, mostly in critically ill patients and immunocompromised hosts [6].

Although carbapenem-resistant *Klebsiella pneumoniae* (CR-Kp) represent a major concern in hospital settings, due to the production of carbapenemases [7], other pathogens, such as *Pseudomonas* spp. and *Acinetobacter* spp., are emerging causes of DTT and difficult-to-eradicate infections, leading to recurrent infections, additional costs and length of hospital stay and dramatic mortality risk [8].

Colistin has been long considered the backbone of therapeutical strategies against XDR GNB; however, the unpredictable pharmacodynamic/pharmacokinetic (PK/PD) properties and the considerable kidney toxicity have forced clinicians to search for alternative antimicrobials or combination regimens in order to increase success rates [9]. Additionally, colistin resistance significantly increased in recent years, causing a further reduction of possible treatment options for XDR GNB infections [10]. In this scenario, cefiderocol represents a novel and very promising therapeutic opportunity.

With the exception of the three randomized non-inferiority clinical trials (the CREDIBLE-CR [11], the APEKS-NP [12] and the APEKS-cUTI [13]), only a few case reports and small case series described the use of cefiderocol in real-life settings [14,15,16], showing encouraging results. Accordingly, the aim of this study is to provide a description of our compassionate use clinical experience, and discuss possible future perspective of this new molecule in the treatment of severe infections in critically ill and immunocompromised hosts.

## 2. Case Series

Overall, 13 patients underwent treatment with cefiderocol. Median (q1–q3) age was 63 (53–69) years, and 11 subjects (84%) were males. Importantly, cefiderocol was used in three specific settings in our series:

(i) 5/13 (38%) critically ill patients with severe lung failure due to underlying SARS-CoV-2 infection;
(ii) 4/13 (31%) post-surgical infections;
(iii) 4/13 (31%) severe infections in immunocompromised patients due to solid organ transplantation (2/4) or hematological malignancy (2/4).

Median time to diagnosis of the infection since hospital admission (or surgical procedures) was 10 (9–21) days. Overall, 10 carbapenem-resistant *A. baumannii* (CRAB), 1 ceftazidime/avibactam-resistant KPC producing *K. pneumoniae* (KPC-Kp) and 2 XDR *P. aeruginosa* (XDR-PA) were isolated from blood cultures (10/13), purulent abdominal drainages (2/13), tracheobronchial aspirates (2/13) and purulent drainage from a neurosurgical site (1/13). In the following paragraphs, the three different groups of patients are thoroughly described.

### 2.1. Critically Ill Patients Due to Severe Lung Failure in the Course of SARS-CoV-2 Infection

All five critically ill patients included in this group were affected by primary central venous catheter (CVC)-CRAB BSI, which occurred during hospitalization: in 3/5 presenting as a septic shock and in 2/5 as a sepsis, with a median Sequential Organ Failure Assessment (SOFA) score of 6 (5–8).

At symptoms onset, 2/5 were mechanically ventilated, while the remaining 3/5 were hospitalized in sub-acute medical wards; all patients were treated with a colistin-based combination therapy and CVC removal, as shown in Table 1; however, after a median of 5 (4–7) days, the regimen was discontinued due to unsatisfactory clinical response, colistin resistance or colistin toxicity with persistence of positive blood cultures. Hence, a subsequent cefiderocol-based therapy, combined with fosfomycin, tigecycline or colistin, was initiated and continued for a median of 8 (5–10) days. Follow-up blood cultures were obtained at 48 h from the initial cefiderocol-based therapy (one set every day for aerobes and anaerobes), documenting complete bacteriemia clearance in all cases. Notably, no cefiderocol-related adverse events were recorded.

In all cases, a full dosage of 2 g of cefiderocol every 8 h was prescribed, excluding patient 5, who was affected by mild/moderate kidney failure due to colistin toxicity. In this case, a dosage of 1.5 g every 8 h was prescribed due to a median creatinine clearance of 45 mL/min, as per the SmPC guideline.

Clinical success, along with microbiological eradication from blood cultures, was achieved in all cases; however, one patient (Pt1) died due to worsening of SARS-CoV-2-related respiratory failure. Importantly, this patient was also affected by Huntington Chorea and immobilization syndrome; hence, he was not suitable for intubation and ICU hospitalization and deceased due to progressive muscular failure despite appropriate non-invasive ventilation. No Ventilator Associated Pneumonia (VAP) was documented by non-invasive upper respiratory lung sampling.

### 2.2. Post-Surgical Infections

Herein, we reported the use of cefiderocol in four male patients who developed severe “difficult-to-treat” gram-negative infections in the immediate post-surgical phases. The general characteristics of patients are reported in Table 2.

As opposed to the previously described group, four types of infections were diagnosed in this case:

(i) A VAP caused by CRAB, in a patient mechanically ventilated post neurosurgical treatment for cerebral hemorrhage (Pt6);
(ii) A CRAB BSI post-coronary angioplasty in a subject hospitalized for myocardial infarction in course of mild SARS-CoV-2 infection (Pt7);
(iii) A neurosurgical wound infection post-parietal bone excision caused by XDR-*P. aeruginosa* (Pt8);
(iv) A tertiary peritonitis with polymicrobial intrabdominal abscesses caused by CRAB, XDR-*E. cloacae complex*, *M. morganii* and ampicillin-resistant *E. faecium* in a patient hospitalized in the ICU (Pt9).

Notably, all patients began cefiderocol-based combination therapy due to unsatisfactory clinical response to previous therapy; in addition, both Pt8 and Pt9 required a concurrent surgical debridement in order to achieve clinical success, which was obtained for all patients. At presentation, the median SOFA score was 3 (0–4). In all cases, a full antibiotic dosage was prescribed since no patient presented an altered kidney or hepatic function.

The duration of compassionate treatment was defined according to current guidelines for different infections, excluding the case of Pt9, whose critical conditions and fever persisted for 8 days after the initiation of cefiderocol; consequently, a prolonged duration of treatment was administered (21 days) until the resolution of intrabdominal abscesses as assessed by ultrasonography.

With the exception of one case (Pt7), where cefiderocol was used in addition to colistin and fosfomycin while awaiting results for sensitivity testing on a CRAB strain, the drug was prescribed in combination with fosfomycin or tigecycline. No adverse events to cefiderocol were recorded.

### 2.3. Severe Infections in Immunocompromised Patients

The last four patients of this series were severely immunocompromised at the time of XDR GNB infection (Table 3). Importantly, two of them were solid organ transplant recipients (heart and liver, respectively), and both were affected by hematologic malignancies; in one case a myelodysplastic syndrome, in the other case an acute myeloid leukemia treated with allogenic stem cells transplantation. Additionally, in these cases, different types of infections were recorded:

(i) Liver abscesses with BSI occurred 6 months after liver transplantation caused by KPC-producing *K. pneumoniae* resistant to ceftazidime/avibactam (Pt10);
(ii) VAP with BSI caused by CRAB in a heart transplant recipient (Pt11);
(iii) BSI due to CRAB in a patient with myelodysplastic syndrome, hospitalized for COVID-19 (Pt12);
(iv) Severe multifocal pneumonia caused by XDR-*P. aeruginosa* in a patient with acute myeloid leukemia who underwent allogenic stem cells transplantation (Pt13).

Importantly, patients in this group presented with a median SOFA score of 6 (4–11); additionally, two of them, Pt11 and Pt12, were hospitalized in intensive care unit at time of infection due to severe general clinical condition.

In all cases, the cefiderocol-based therapy was initiated due to unsatisfactory response to previous treatments and/or failure in microbiological eradication as assessed by biological samples. Herein, a prolonged treatment (at least 10 days) was administered in all patients, considering their impaired immune response, in order to reduce the risk of infection recurrence. Differently from previous cases, a combination with colistin plus an additional third drug was prescribed, excluding the case of XDR-*P. aeruginosa* pneumonia (due to the limited penetration of colistin in lung tissues).

For all patients, the 2 g T.I.D. cefiderocol regimen was prescribed, including Pt11, who was undergoing high effluent (4 L/hr) rate continuous-renal-replacement-therapy (CRRT) and a full dose of the antibiotic was requested according to the manufacturer sheet.

Noteworthy, a complete microbiological eradication was achieved in all cases, although two patients deceased within 30 days. In one case, the unfavorable outcome was caused by the worsening of COVID-19 pneumonia (Pt12), without the occurrence of any new secondary infection, as documented by multiple negative microbiologic sampling, including upper and lower respiratory tract sampling. In the other case (Pt11), the patient suffered from multiple hospital complications, including gastroenteric bleeding due to congenital coagulopathy, a CVC-related BSI due to methicillin-resistant *Staphylococcus epidermidis* and a hepatosplenic invasive candidiasis due to *C. parapsilosis*, a hemophagocytic syndrome. Moreover, he also suffered from *C. difficile* colitis during the course of cefiderocol-based combination therapy.

## 3. Discussion and Future Perspectives

This is among the most extensive case series describing the real-life use of cefiderocol for the treatment of severe XDR gram-negative bacterial infections, along with the multicentric experience by Bleibtreu and colleagues [17] and the monocentric experience of Falcone et al. [18]. In addition, this series enrolled “difficult-to-treat” patients, due to their important comorbidities, severe clinical conditions requiring ICU admission or deep immunocompromission. Indeed, the hardest treatment challenge is often fought in these settings, where multiple variables could severely influence the patient outcome; consequently, pathogens causing the infections, PK/PD characteristics of antibiotics and the need of combination therapy should be carefully considered along with patients’ features, so as to obtain the correct place in the therapy of different drugs and achieve optimal results.

Importantly, beside the improvement of different therapeutic options, infection control remains the first and most important intervention to hamper the spreading of multidrug resistant organisms and reduce the morbidity of these infections. In the following paragraphs, the microbiological, pharmacological and possible clinical uses of cefiderocol are discussed.

### 3.1. Spectrum of the Activity of Cefiderocol Against “Difficult-To-Treat” Bacteria

Cefiderocol has a significant antibacterial activity against Gram-negative bacteria such as Enterobacterales and non-fermenting bacilli [1]. Indeed, it shows minimal inhibitory concentration (MIC) values ≤2 μg/mL against *Acinetobacter* spp., *Klebsiella* spp. and *Pseudomonas* spp. [19]. On the other hand, cefiderocol demonstrates a weaker activity against aerobic Gram-positive or anaerobic pathogens. While the mechanism behind the inefficacy against aerobic Gram-positive bacteria has not been sufficiently investigated, for anaerobic microorganisms, it seems to be partially explained by a lower reliance on the siderophore-iron transporter system for growth under anaerobic conditions [20,21].

In Gram-negative bacteria, cefiderocol is able to penetrate into the periplasmic space and overcome the most common mechanisms of β-lactam resistance among Gram-negative microorganism, including porin deficiency, up-regulation of efflux pump expression and the production of β-lactamases. Indeed, it has an increased stability against hydrolysis by various types of β-lactamases, including both serine-based (KPC, OXA) and metallo-type (VIM, IMP, NDM) carbapenemases. Cefiderocol also presents antibacterial effectiveness against AmpC-overproducing strains of *P. aeruginosa* and *E. cloacae*, low affinity for chromosomal AmpC β-lactamases and low induction [22].

Importantly, new antimicrobial agents recently approved for treatment of Gram-negative bacilli, such as new β-lactam/β-lactamase inhibitors (ceftazidime/avibactam, ceftolozane/tazobactam, meropenem/vaborbactam), show gaps of activity against some carbapenemases; thus, cefiderocol could play an important role in effective therapy against “difficult to-treat” Gram-negative bacteria, particularly MBL-producing Enterobacterales or ceftazidime/avibactam resistant KPC-*K. pneumoniae*, as well as XDR-*P. aeruginosa* and CRAB [23,24,25].

### 3.2. Pharmacologic Aspects of Cefiderocol

Cefiderocol is a third-generation cephalosporin antibiotic class and is the first siderophore antibiotic approved by the FDA [26]. It is an injectable siderophore cephalosporin with potent broad-spectrum activity against aerobic MDR GNB, including the three pathogens declared as critical priority by the WHO: *A. baumannii*, *P. aeruginosa* and Enterobacterales resistant to carbapenems [27]. In addition, it displays in vitro activity against bacteria expressing enzymes that confer resistance, and therefore, those that are difficult to eradicate, such as ESBLs, AmpC, serine and metallo-beta lactamases.

The structural characteristics of cefiderocol show similarities with both ceftazidime and cefepime. Particularly, the addition of a catechol moiety on the C-3 side chain, with iron chelating activity mimics siderophore molecules produced by bacteria, conferring cefiderocol’s resistance to hydrolysis induced by β-lactamases. After the iron chelation, cefiderocol is actively transported across the bacterial outer membrane into the periplasmic space, through specific iron transport channels [28]. These channels allow cefiderocol to move easily within the cell wall, unlike other β-lactam antibiotics which can only act outside of this membrane and through other membrane permeability structures.

In vitro studies have shown that cefiderocol is 10 to 100 times more stable to different types of carbapenemases compared to ceftazidime. As opposed to other novel antibiotics and antibiotic/inhibitor combinations, cefiderocol also displays excellent in vitro activity against most of the class A, B, C and D β-lactamases of the Enterobacterales species [29].

In phase I studies, cefiderocol demonstrated a linear pharmacokinetics in the dose range of 100 mg to 4000 mg, a renal-type excretion, a 2–3 h elimination half-life and 58% protein binding in human plasma. Cefiderocol is a time-dependent cephalosporin: based on animal PK models, a ***f***T > MIC of 75% of the dosing interval was selected as the target for cefiderocol. During this period, the free-drug concentration exceeding the minimum inhibitory concentration (ƒT/MIC) for the strains bacterial with a MIC ≤ 4 μg/mL can be reached with a regimen of 3-h infusion of 2 g every 8 h. Markers of renal function are the most influential covariates for the cefiderocol pharmacokinetics for patients with renal failure or increased renal clearance (ARC) [29].

Dose adjustment is recommended for patients with impaired renal function; moreover, in patients with ARC showing a creatinine clearance > 120 mL/minute, a more frequent dosing regimen was planned to achieve the target fT > MIC, i.e., 2 g every 6 h. The single and multiple doses of cefiderocol tested were well tolerated in both healthy subjects and those with renal insufficiency. Furthermore, in healthy subjects, neither QT interval prolongation nor drug–drug interaction via organic anion transporters were observed [28,29].

The most commonly reported adverse reactions were diarrhea (8.2%), vomiting (3.6%), nausea (3.3%) and cough (2%), as well as rash including macular rash, maculo-papular rash, erythematous rash and drug eruption and infusion site reactions including infusion site pain, injection site pain, infusion site erythema and injection site phlebitis. Less common events were hypersensitivity reactions, including skin reactions and pruritus.

The CREDIBLE-CR study demonstrated the cefiderocol efficacy and safety compared to Best Available Therapy (BAT) in the treatment of severe infections caused by carbapenem-resistant Gram-negative bacteria: this was a randomized Phase III, open label, multicenter, pathogen-focused and descriptive study [11].

In 71% of the patients assigned to BAT, the treatment was represented by combination therapy, with approximately 28 different combination regimens used, whereas in the 80% of patients treated with cefiderocol, this drug was used alone. Lastly, in 66% of BAT regimens, colistin backbone was employed for the treatment of severe infections caused by multidrug-resistant gram-negative bacteria, against which no other antibiotics proved their efficacy.

Colistin is an antibiotic belonging to the cationic glycopeptide antibiotics class and can be deemed as bacterial cell membrane surfactant: it acts against Gram-negative bacteria, by binding to the anionic component of the lipopolysaccharide membrane, resulting in the death of the bacteria [30]. Over time, bacteria could develop resistance mechanisms by modifying the lipid fraction of the lipopolysaccharide outer membrane: these changes positively charge the cell surface, which lacks affinity for positively charged polymyxins [31]. Therefore, in case of severe gram-negative infections with few therapeutic alternatives showing resistance to colistin, the use of cefiderocol proved decisive.

The results of the CREDIBLE-CR study [11] highlighted cefiderocol’s efficacy and safety in Gram-negative infections in a highly heterogeneous population of patients, who frequently have complex comorbidities. Clinical and microbiological outcomes were generally similar between cefiderocol and BAT. An imbalance of risk factors in the subgroup of patients with *Acinetobacter* spp. infections likely contributed to the difference in mortality observed between the two treatment arms. No differences in mortality were observed in patients with *P. aeruginosa*- or *Enterobacterales*- sustained infections without *Acinetobacter* spp. co-infections. None of the deaths were attributed to cefiderocol-related adverse events, as reviewed by the participating investigators and regulatory bodies assessing the mortality imbalance. Still, although not fully elucidated and currently under investigation, the CREDIBLE-CR resulted in a higher mortality in the cefiderocol group if compared with the BAT group. Therefore, the Food and Drug Administration included a warning box on this topic, recommending cefiderocol as a first line treatment only for complicated urinary tract infections.

### 3.3. The Role of Combination Therapy

The role of combination therapy for the treatment of severe XDR gram-negative infections has long been debated [31]. Overall, in clinical practice, when a multidrug resistant gram-negative pathogen sustained infection is suspected, combination therapy is usually preferred in order to increase the possibility of initiating at least one effective treatment against the underlying cause of infection, especially in course of severe infections. However, definitive data are still lacking. In addition, beside the main benefit mostly driven by the increased spectrum of activity, combination therapy could have other advantages; for instance, in case of CRAB BSI, studies suggested a possible increased survival rate with combination therapy if compared with monotherapy [32], with beneficial effects of some combinations over others [33]. Indeed, several advantages could derive from the use of combination therapies: (i) synergistic effects with faster bacterial clearance; (ii) reduced emergence of resistant strains; (iii) broad spectrum activity (particularly useful in polymicrobial infections) [31].

Firstly, upon combination of different classes of antibiotics, a synergistic effect can be obtained, determining a greater bactericidal action, since the therapeutic action is greater than the sum of each drug. A synergistic effect could determine a reduction in length of symptoms and possibly overall duration of antibiotic therapy [32]. Furthermore, a combination therapy could overcome antimicrobial resistance, allowing the use of agents against which bacteria had become resistant [34]. Indeed, several studies have compared the use of monotherapy vs combination therapy in difficult-to-treat infections caused by MDR pathogens. For example, Lenhard and colleagues demonstrated that the use of colistin + meropenem against XDR *A. baumannii* (colistin, meropenem and ampicillin/sulbactam resistant) did not lead to eradication of the infection, whereas addition of ampicillin/sulbactam made it possible [35]. Bulman and colleagues showed a similar effect on mcr1 + blaNDM + *E. coli* using colistin, aztreonam and amikacin alone or in combination. The bacterium was resistant to each single agent, but susceptible and completely eradicated by combination therapy [36].

Secondly, another advantage of combination therapy is the reduction of onset of resistance. The spontaneous development of resistance often occurs by chance. Therefore, the use of more antibiotics reduces the risk of concurrent selection of spontaneous resistance. However, the development of resistance does not always target a single molecule, but in some cases, a cross-resistance to multiple classes of antibiotics may develop. This is the case of efflux pumps and porin mutations that confer resistance to multiple agents [37,38]. Moreover, recent experiences also explored this effect in vivo: the initiation of combination therapy could possibly reduce the risk of developing a subsequent multidrug-resistant Gram-negative BSI or fungemia in patients with BSI, if compared with those treated with monotherapy [39].

A third aspect is represented by the broader spectrum of activity that is associated with increased possibility of initiating an empirical antibiotic regimen with at least one active drug, providing adequate coverage for potential MDR pathogens, especially in settings with high rates of antimicrobial resistance, thus causing a decreased mortality rate [40,41]. However, recent studies demonstrated that in settings with lower rates of antimicrobial resistance, expanding the spectrum of activity of initial antimicrobial therapy did not reduce mortality but increased toxicity [42].

On the other hand, combination therapy surely has some disadvantages, such as: (i) increasing toxicity due to pharmacokinetic interactions (i.e., vancomycin plus piperacillin/tazobactam can cause an increase of acute kidney failure) [43]; (ii) increasing the risk of C. difficile infections; (iii) risk of fungal overgrowth and invasive fungal infections; (iv) need of dedicated catheter accesses (complicating nursing management and risk of catheter-associated complications). Cefiderocol-based combination therapy has not been studied yet taking these issues into account.

A few in vitro reports suggested a potential beneficial effect of combination therapies, especially for “difficult-to-treat” pathogens [44]; until further data are available, clinicians need to outweigh the risks and benefits of cefiderocol, and consideration should be given to combination therapy.

However, due to the pharmacodynamic characteristics of cefiderocol, the choice of a combination therapy appears to be the safest, in order to avoid resistance development and to exploit a potential synergistic/additional effect. Therefore, the choice of “companion drug(s)” need to be tailored according to the involved pathogen(s) in order to obtain the maximum efficacy. Overall, the use of a second drug displaying full activity against the isolated pathogen should be encouraged, at least until future studies will demonstrate a clear synergistic effect between cefiderocol and other antibiotics. For instance, in our experience, cefiderocol displayed a high efficacy against *P. aeruginosa* when combined with Fosfomycin, while colistin or a high dose of tigecycline were preferred against CRAB. Conversely, our experience was limited to treating ceftazidime/avibactam-resistant KPC-*K. pneumoniae*; however, the definitive treatment regimen was built with fosfomycin due to its reduced toxicity. Further studies are certainly warranted on this topic.

### 3.4. Place in Therapy in Critically Ill Patients

In our series, the group of critically ill patients was mainly composed of subjects suffering of severe acute respiratory failure with underlying COVID-19 disease. However, a few patients, enrolled in the post-surgical group or the immunocompromised group, were also critical and hospitalized in intensive care units (ICUs). Interestingly, these categories are very different for multiple reasons, and the spectrum of “critically ill patients” is remarkably wider; therefore, certain considerations can be applied only in our setting.

Overall, it should be acknowledged that within the group of “critically ill,” only patients with CVC-related BSI by CRAB were enrolled. Particularly, excluding the case of (Pt1), all patients were known to be colonized by CRAB, and a colistin-based therapy was immediately started at symptoms onset, along with the vascular device removal when the blood culture was positive. Consequently, the use of cefiderocol was restricted to the later phases of the infection, when patients were already hemodynamically stabilized, and the device removed. Still, blood cultures remained persistently positive, and the drug was used to achieve complete resolution of bacteremia. Interestingly, all patients enrolled obtained negative blood cultures within 48 h from therapy initiation, although one subject (Pt1) deceased a few days later due to COVID-19-related complications along with the detrimental effects of sepsis, as the anti-CRAB treatment was started with a 48 h delay due to his unknown colonization status. Indeed, one of the main concerns in ICU is the appropriateness of initial antibiotic therapy upon diagnosis of septic shock [45], whereby treatment delay is markedly associated with increased risk of death. Accordingly, international sepsis management guidelines [46] suggested to use a wide spectrum antibiotic therapy, possibly within 1 h from the clinical diagnosis of septic shock [47], in order to reduce mortality. However, in the context of patients colonized by multiple MDR or XDR bacteria, such as those hospitalized in ICU for a long time, selecting an early empirical antibiotic therapy with at least one active drug against the causative pathogen could be challenging without mixing multiple drugs.

A future place in the therapy of cefiderocol could, eventually, be as initial antibiotic therapy in patients colonized by XDR Gram-negative pathogens with limited treatment options or colonized by multiple pathogens with different spectrums of sensitivity, in case of high risk of sepsis caused by those resistant strains. Indeed, by exploiting its high bactericidal and broad spectrum of activity [21], clinicians could provide an early complete coverage against Gram-negative bacteria. However, the use of cefiderocol in septic shock should be discouraged until further evidence will suggest its effectiveness. Indeed, according to the FDA label and CREDIBLE-CR study [11], cefiderocol in this setting has been related to a higher mortality compared to the best available therapy, although the precise explanation has still not been elucidated. Yet, the advantages of this choice should be balanced with the risk of developing resistance, particularly in case of inappropriate use, or in case of inadequate compliance with PK/PD [48,49], and therefore, it should be reserved for the treatment of severe infections in selected patients.

Moreover, our experience confirmed the remarkable efficacy of cefiderocol in treating nosocomial pneumonia: notably, three patients (Pt6, Pt11, Pt13) successfully eradicated the lung infections after failing the conventional treatment based on currently available drugs. This result is in line with previous reports suggesting the efficacy of cefiderocol for the treatment of VAP [18] caused by different carbapenem-resistant Gram-negative bacteria.

Of note, VAP still remains a difficult-to-treat infection, particularly when the underlying cause are XDR pathogens; indeed, treatment strategies are controversial, often involving inhaled antibiotics [50], along with intravenous administration, although their role is not well established yet [51]. On the other hand, the higher efficacy of Cefiderocol-based regimens in the reported series can be possibly explained by its convenient PK/PD properties in lung tissues [52], along with its linear killing kinetics of resistant bacteria, including Enterobacterales, *P. aeruginosa*, *A. baumannii* and *S. maltophilia* [52].

Finally, cefiderocol demonstrated its efficacy for the treatment of complicated intrabdominal infections (cIAI) caused by multiple pathogens. According to previous reports [53,54], in one case (Pt9), we decided to start a cefiderocol plus a high dose of daptomycin, tigecycline and fosfomycin to treat the infection along with an appropriate source control procedure. The main driver of this combination strategy was the sensitivity pattern of bacteria and the high tolerability of drugs selected, if compared with colistin and aminoglycosides, or vancomycin, considering the prolonged planned duration of the therapy (21 days). As a fact, in the other cIAI case in our series (Pt10), colistin was discontinued early due to toxicity. Accordingly, a possible further place in therapy of cefiderocol could be represented by long-lasting therapies for complicated infections due to its higher safety when compared with other second line drugs for XDR pathogens.

Future studies should explore the best clinical use for this complex setting, as often, these patients are at risk of increased mortality for many different causes. Future research should preferably use composite efficacy outcomes (microbiological eradication, time to signs and symptoms resolution, occurrence of adverse events etc.) along with crude mortality to appropriately evaluate the efficacy of cefiderocol in these settings.

### 3.5. Place in the Therapy of Immunocompromised Hosts

Within the context of DTT infections, a special focus should be made on immunocompromised patients, such as solid organ transplant recipients and those affected by hematological malignancies.

Importantly, solid organ transplant recipients are at high risk of bacterial infections, especially during the first month after transplantation, including donor-derived or pre-existing recipient infections [55,56], which represent one of the main causes of mortality and graft failure [57]. Not surprisingly, nosocomial infections and surgical complications caused by DTT bacteria, above all carbapenem-resistant Gram-negatives and non-fermentative Gram-negatives, play a major role in terms of incidence [58] and risk of mortality [59].

The same issue can be highlighted among patients affected by solid tumors and hematologic malignancies; indeed, in the era of multidrug resistance, the occurrence of severe infections in this setting is associated with extremely high mortality [60,61]. Finally, the category of patients affected by autoimmune diseases, exposed to immunosuppressive drugs, are at higher risk of secondary infections and need to be included in future studies exploring possible management and treatment options tailored according to their particular conditions [62]. Additionally, in the setting of immunocompromised hosts, multiple factors contribute to the severity of infections, such as neutropenia, long-term use of immunosuppressive drugs, exposure to empirical broad-spectrum antibiotic, indwelling catheters, chemotherapy-induced mucositis and intestinal bacterial translocation [63]. Moreover, the occurrence of further physio-pathological changes, such as cachexia, hypoalbuminemia and augmented renal function, may negatively affect antimicrobial pharmacokinetics, increasing the risk of treatment failure and occurrence of antibiotic resistance [64]. Considering all these factors, the need of highly effective antimicrobials which may overcome the reduced immune function of these patients is highly warranted.

Currently, the backbone of carbapenem-resistant anti Gram-negative therapy is represented by colistin, which expresses its therapeutic efficacy according to efficacy parameters determined by the Concentration-Dependent and Time-Dependent pharmacokinetic index (AUC/MIC) [65]; however, the aforementioned metabolic changes in immunocompromised patients cause larger volumes of distribution with lower drug plasmatic concentrations and highly unpredictable efficacy, eventually requiring the need of administering higher doses of colistin to achieve proper bactericidal activity, and consequential important risk of nephrotoxicity.

These conditions raise the risk of failure in treatment of infections caused by XDR GNB, particularly when mortality and microbiologic eradication are compared between antimicrobials with linear kinetics, such as cephalosporins, and other active antibiotics [66]. However, the use of penicillins should also be improved in this setting; according to recent studies, penicillins’ and cephalosporins’ Time > MIC target should be closer to 100% in order to achieve higher chances of efficacy on GNB infections in immunosuppressed hosts, while in immunocompetent, a T > MIC target of 50–70% is considered adequate to ensure standard efficacy [67].

Overall, in our experience, cefiderocol-based combination therapy was used in all cases for the treatment of severe infections upon failure with previous antibiotic regimens. Importantly, we recorded two cases of mortality in among immunocompromised hosts treated in our series: in both cases (Pt11 and Pt12), the negative outcome occurred following microbiological eradication. In one case (Pt11, heart transplant recipient), death was due to concurrent secondary complications and worsening of clinical conditions deriving from the underlying CRAB infection, whereas in the other case (Pt12, affected by myelodysplastic syndrome), death was due to worsening of COVID-19 disease.

In this setting, with highly fragile patients, an early effective treatment with lower risk of treatment failure, toxicity and prolonged duration of infection could significantly impact overall survival. In this sense, cefiderocol, as well as other cephalosporins, represents a valuable therapeutic backbone for immunocompromised hosts with severe XDR GNB infections.

Starting from these considerations, a possible future place in therapy of cefiderocol, along with the treatment of severe infections caused by CRAB, XDR *P.aeruginosa* or metallo-beta-lactamase producing Enterobacterales, may represent an option as empirical therapy for the management of febrile neutropenia in hematological patients colonized by these pathogens or in the perioperative prophylaxis of donor-derived infections (DDIs) in transplant recipients when the donor was colonized or infected by XDR GNB [68]. For instance, recent studies suggested the efficacy and safety of ceftazidime/avibactam or ceftolozane/tazobactam as empiric therapies of febrile neutropenia in hematological patients colonized by multidrug-resistant organisms [69,70]; however, using this approach, a rapid, programmed and structured de-escalation approach needs to be implemented in order to reduce the risk of developing resistance as well as to preserve the activity of these new antibiotics. Similarly, as discussed before, an important role of new cephalosporins will be played in the treatment of XDR GNB that caused DDIs [68]. A recent experience with ceftazidime/avibactam as rescue treatment for recipients receiving a solid organ transplant, whereby the organ donor was colonized by carbapenem resistant GNB, demonstrated a valuable activity of these cephalosporins in this setting [71]. A similar cefiderocol-based strategy may be proposed in the future, expanding the reservoirs of transplantable organs, including those colonized by XDR pathogens, such as CRAB, XDR *P. aeruginosa* or metallo-beta-lactamase producing Enterobacterales. At any rate, the complexity of infection management in immunocompromised hosts requires further extensive studies.

## 4. Materials and Methods

### 4.1. Patients and Treatments

We prospectively collected data of patients with XDR GNB infections who were treated with cefiderocol in the tertiary-care University Hospital of Bari (Azienda Ospedaliera Universitaria Policlinico di Bari) from 1 September 2020 to 20 April 2021. As a University Hospital, our Center took part to the Early Access Program of Shionogi & Co. Ltd. (closed on 26 April 2021). Consequently, each cefiderocol treatment was requested after the approval of our Ethical Committee in compassionate use. Finally, each treatment was furnished by the Inceptua Group (https://www.inceptua.com/) after submitting a request through their website. Patients were selected if they had documented infections due to fermenting or non-fermenting GNB resistant to carbapenems and susceptible to cefiderocol (MIC ≤ 2 μg/mL) and experienced clinical failure and/or severe adverse events from previous antibiotic regimens.

Each enrolled patient was affected by severe infections/multidrug resistant organism(s) and underwent revision every 48–72 h by an Infectious Diseases specialist during treatment. Clinical failures/cures were assessed according to the achievement or not of at least one item, among the following, at each programmed re-evaluation: (i) clinical or microbiological improvement, (ii) persistence of signs and symptoms of infection, (iii) worsening of clinical condition or (iv) dissemination of the infection in course of treatment.

As stated by our internal protocol, patients with bloodstream infections repeated follow-up blood cultures after 48 h from the initiation of targeted antimicrobial treatment, consisting of one culture set/day for both aerobes and anaerobes for three consecutive days.

Secondary infections, including VAP, tracheobronchitis, pneumonia [72], CVC-related BSI [73], surgical site infections [74] and intrabdominal infections [75], were defined according to current guidelines.

Clinical conditions of patients with COVID-19 were defined as mild/moderate, severe or critical according to the current guidelines [76]:

- Mild/Moderate, if they had clinical signs of pneumonia (fever, cough, dyspnea, fast breathing) but no signs of severe pneumonia, including SpO2 ≥ 90% on room air;
- Severe, if they had signs of pneumonia (fever, cough, dyspnea, fast breathing) plus one of the following: respiratory rate > 30 breaths/min; severe respiratory distress; or SpO2 < 90% on room air;
- Critical, if a diagnosis of acute respiratory distress syndrome (ARDS) was made.

Cefiderocol was administered as a 3-h IV infusion at a standard dose of 2 g, diluted in at least 100 mL of saline solution, intravenously every 8 h, with adjustments for renal impairment made according to the manufacturer’s recommendations (SmPC). All patients were prospectively followed up to day 30 or until death occurred. Recovery was defined as a composite endpoint: survival, resolution of signs and symptoms of infection and absence of recurrent infection. Microbiological eradication was defined as negative blood culture in case of BSI or negative culture result of sampling of previous site of infection, when possible, after the end of the therapy.

### 4.2. Bacterial Strains

All Gram-negative isolates from patients included were defined as multidrug resistant (MDR), extensively drug resistant (XDR) or pandrug resistant (PDR) according to the following definitions [77]:

MDR: non-susceptible to ≥1 agent in >3 antimicrobial categories.

XDR: non-susceptible to ≥1 agent in all but ≤2 categories.

PDR: non-susceptible to all antimicrobial agents listed.

### 4.3. Sampling Process

Samples were collected for microbiological assessment before starting empirical cefiderocol therapy. According to the current guidelines, blood cultures were performed by collecting 20–30 mL of blood per culture set. Two bottles per set were used and immediately placed into a BACT/ALERT^®^ 3D instrument (Biomerieux Inc., France). Positive aerobic blood cultures were subcultured on MacConkey agar, CNA blood agar, Sabouraud dextrose agar, mannitol-salt agar and Chocolate agar and incubated aerobically at 37 °C for 24 h

Tracheobronchial aspirates and purulent drainages were directly inoculated and incubated aerobically at 37 °C for 24 h on MacConkey agar, CNA blood agar, Sabouraud dextrose agar, mannitol-salt agar and Chocolate agar. Moreover, purulent drainages were also transferred to enriched brain heart infusion (BHI) broths and incubated at 37 °C for 24 h. Identification was performed using VITEK-MS (Biomerieux Inc., France) according to the manufacturer’s instructions.

### 4.4. Antibiotic Susceptibility Testing

Susceptibility testing was performed using the Vitek^®^ 2 automated system (biòMerieux, France). In addition, cefiderocol susceptibility testing was performed both with disk diffusion (Liofilchem srl) and broth dilution (SensititreTM, Thermo Fisher Scientific). MIC values were interpreted according to the clinical breakpoints established by the European Committee on Antimicrobial Susceptibility Testing (EUCAST, 2020).

## 5. Limitations

Overall, this study has some limitations requiring acknowledgement. Indeed, we were unable to perform bacterial genetic typing; hence, a more precise bacterial description, including mechanisms of resistance of all pathogens and precise MIC estimation for different antibiotics, are lacking. Moreover, therapeutic drug monitoring of antimicrobials, in particular colistin, cefiderocol and fosfomycin, was not available in our center. To conclude, as a small retrospective case series, our results are not generalizable.

## 6. Conclusions

Cefiderocol will provide a new tool against “difficult-to-treat” GNB, expanding the clinician armamentarium. Because of its potential, it is essential to increase real-life data and deepen the knowledge on its correct place in therapy in both empirical and targeted strategies, avoiding at the same time the emergence of resistance. In addition, multiple settings, including critically ill, post-surgical and immunocompromised hosts, will benefit from studies tailored according to their specific characteristics.

## Figures and Tables

**Table 1:** Characteristics of patients of Group 1, classified as “critically ill.”.

	Age (Year)	Sex	Cause of Hospitalization	Underlying Diseases	Ward	Pathogen	Type of Infection	Initial Therapy (*)	Cause of Failure	Cefiderocol Based Therapy (*)	Outcome	Outcome at 30 Days
Pt1	68	M	COVID-19	Huntington Chorea, Immobilization syndrome, Severe COVID-19 disease	Internal Medicine, COVID Unit	CRAB	CVC-related BSI with Septic Shock	CST, TG, FOF (4)	Unsatisfactory clinical response	FDC, FOF, TGC (5)	Microbiological Eradication	Death†
Pt2	62	F	COVID-19	Fibromyalgia	Intensive Care Unit	CRAB	CVC-related BSI with Septic Shock	MEM, CST (7)	CST resistance	FDC, CST, MEM (13)	Recovery	Success
Pt3	69	M	COVID-19	Hypertension, Diabetes	Intensive Care Unit	CRAB	CVC-related BSI with Septic Shock	MEM, CST (10)	Unsatisfactory clinical response	FDC, CST (10)	Recovery	Success
Pt4	78	M	COVID-19	Hypertension, COPD, Diabetes	Internal Medicine, COVID Unit	CRAB	CVC-related BSI with Sepsis	MEM, CST, TG (2)	Unsatisfactory clinical response	FDC, TGC (8)	Recovery	Success
Pt5	75	F	COVID-19	Diabetes	Infectious Diseases	CRAB	CVC-related BSI with Sepsis	MEM, CST, FOF (5)	CST toxicity	FDC, FOF (5)	Recovery	Success

Abbreviations: Pt, patient; CRAB, Carbapenem Resistant A. baumannii; CR-Kp, Carbapenem-Resistant Klebsiella pneumoniae; CST, Colistin; DAP, Daptomycin; FDC, Cefiderocol; FOF, Fosfomycin; SAM, Ampicillin/Sulbactam; TEC, Teicoplanin; VAN, Vancomycin; VAP, Ventilator Associated Pneumoniae; XDR, Extensive Drug Resistant; † Microbiological eradication, death from COVID-19; (*), (duration in days).

**Table 2:** Characteristics of patients in Group 2, with a post-surgical infection.

t	Age (Year)	Sex	Cause of Hospitalization	Underlying Diseases	Ward	Pathogen	Type of Infection	Initial Therapy (*)	Cause of Failure	Cefiderocol Based Therapy (*)	Outcome	Outcome at 30 Days
Pt6	38	M	Dyspnea post orotracheal intubation for cerebral hemorrhage	Hypertension, Pulmonary Embolism	Thoracic Surgery	CRAB	VAP	CST, FOF, TGC (4)	Unsatisfactory clinical response	FDC, FOF, TGC (9)	Recovery	Success
Pt7	70	M	PTCA due to myocardial Infarction in course of COVID-19	Mild COVID-19, Diabetes, Ischemic heart disease, Dyslipidemia	Internal Medicine, COVID Unit	CRAB	Bloodstream infection	MEM, CST, FOF, SAM (2)	Unsatisfactory clinical response	FDC, CST, FOF (8)	Recovery	Success
Pt8	64	M	Neurosurgical wound Infection	Previous drainage of post-traumatic subarachnoid hematoma, Hypertension, Iatrogenic hypothyroidism	Infectious Diseases	*P. aeruginosa* XDR	Neurosurgical Wound Infection	CST, FOF (5)	Unsatisfactory clinical response	FDC, FOF (10)	Recovery	Success
Pt9	25	M	Subocclusion and volvulus treated with gut surgical resection	Colostomy, Hip and Arm fracture	Intensive Care Unit	Polymicrobial **	Perihepatic Abscess, Septic Shock	MEM, TGC, DAP, FOF (5)	Unsatisfactory clinical response	FDC, TGC, DAP, FOF (21)	Recovery	Success

Abbreviations: Pt, patient; CRAB, Carbapenem Resistant *A. baumannii*; CR-Kp, Carbapenem-Resistant *Klebsiella pneumoniae*; CST, Colistin; FDC, Cefiderocol; LZD, Linezolid; MEM, Meropenem; SAM, ampicillin/sulbactam; VAP, Ventilator Associated Pneumoniae; XDR, Extensive Drug Resistant; (*), (duration in days); PTCA, percutaneous transluminal coronary angiography. **, CRAB, MDR-*E. cloacae complex*, *M. morganii*, Ampicillin-resistant *E. faecium.*

**Table 3:** Characteristics of patients of Group 3, with immunocompromised patients.

	Age (Year)	Sex	Cause of Hospitalization	Underlying Diseases	Ward	Pathogen	Type of Infection	Initial Therapy (*)	Cause of Therapeutic Failure, Day	Cefiderocol Based Therapy (*)	Outcome	Outcome at 30 Days
Pt10	60	M	Sepsis	Hepatic transplantation for HBV-related cirrhosis and HCC, Previous ischemic heart disease	Gastroenterology	CR-Kp (KPC)	Hepatic Abscess, Bloodstream infection	TGC, CZA, CST (3)	Unsatisfactory clinical response	FDC, TGC, CST (17), then FDC, FOF (11) *	Recovery	Success
Pt11	43	M	Myocardial Infarction and cardiogenic shock, Arrhythmic storm, Acute pulmonary edema	Heart transplantation, Hepatic failure, Renal failure in CRRT	Cardiosurgical Intensive Care Unit	CRAB	VAP, Bloodstream infection	CST, MEM, DAP, TGC (12)	Unsatisfactory clinical response	FDC, TGC, CST, FOF (16)	Microbiological Eradication	Death ‡
Pt12	57	M	COVID-19	Myelodysplastic syndrome, Hypertension, Basedow’s disease	Intensive Care Unit	CRAB	Bloodstream infection	MEM, CST (3)	Unsatisfactory clinical response	FDC, CST (12)	Microbiological Eradication	Death †
Pt13	68	M	Pneumonia	Acute Myeloid Leukemia, Chronic Kidney Disease, Hypertension	Hematology	*P. aeruginosa* XDR	Pneumonia	CST, MEM, FOF (10)	Unsatisfactory clinical response	FDC, FOF (10)	Recovery	Success

Abbreviations: Pt, patient; TGC, Tigecycline; CZA, Ceftazidime/Avibactam; GEN, Gentamycin; CRAB, Carbapenem Resistant *A. baumannii*; CR-Kp, Carbapenem-Resistant *Klebsiella pneumoniae*; CST, Colistin; FDC, Cefiderocol; LZD, Linezolid; MEM, Meropenem; VAP, Ventilator Associated Pneumoniae; XDR, Extensive Drug Resistant; HBV, hepatitis B virus; HCC, hepatocellular carcinoma; CRRT, continuous-renal-replacement-therapy. *, Changed for toxicity (Decreased renal function, Bilirubin increased). †, Microbiological eradication, death from COVID-19. ‡ Microbiological eradication, death from subsequent new infections. (*), (duration in days).

## Data Availability

Data available on request from the corresponding author on reasonable request.
